# Acute Cyanide Poisoning: Hydroxocobalamin and Sodium Thiosulfate Treatments with Two Outcomes following One Exposure Event

**DOI:** 10.1155/2015/217951

**Published:** 2015-10-12

**Authors:** Andrew Meillier, Cara Heller

**Affiliations:** Department of Medicine, Temple University Hospital, Philadelphia, PA 19140, USA

## Abstract

Cyanide is rapidly reacting and causes arrest of aerobic metabolism. The symptoms are diffuse and lethal and require high clinical suspicion. Remediation of symptoms and mortality is highly dependent on quick treatment with a cyanide antidote. Presently, there are two widely accepted antidotes: sodium thiosulfate and hydroxocobalamin. These treatments act on different components of cyanide's metabolism. Here, we present two cases resulting from the same source of cyanide poisoning and the use of both antidotes separately used with differing outcomes.

## 1. Introduction

Cyanide has well known adverse effects, some of which are rapidly fatal, documented as early as 1679 [1]. Exposure to cyanide ions can occur through inhalation, skin absorption, and ingestion or even through metabolism. Most commonly, cyanide is inhaled from the thermal breakdown of synthetic compounds during residential or industrial fires [1, 2]. Once internalized, cyanide binds to cytochrome oxidase a_3_, a terminal complex in the electron transport chain [3]. This process prevents aerobic metabolism, which results in diffuse clinical symptoms such as dizziness, headache, weakness, and tachypnea, with progression to seizures, paralysis, and coma [4, 5]. Typically, cyanide toxicity treatment is initiated when there is high clinical suspicion of exposure. There are presently two leading antidote treatments, hydroxocobalamin and sodium thiosulfate, which have been mainly described in case reports and retrospective and prospective studies to demonstrate their functional effectiveness in cyanide poisoning [6, 7]. Here, we present two case reports following the same initial cyanide exposure with two distinct antidote treatments.

## 2. Case Report 1

Patient is a 41-year-old male with past medical history of hypertension who presented following cardiac arrest. The patient was found to be unconscious in a metal chrome plating shop for undetermined duration of time near another unconscious male (Case #2). Emergency medical services arrived and found the patient in asystole and started advanced cardiac life support protocol. Intubation occurred on the field. The resuscitation efforts occurred for 45 minutes while the patient was being transported to an outside hospital. Following return to spontaneous circulation, the patient was hypotensive requiring dopamine and norepinephrine. Due to the work environment, the patient received a cyanide antidote kit (sodium thiosulfate 12.5 grams, sodium nitrite 300 mg) 14 minutes after arrival at the emergency department. Once the patient was hemodynamically stable, he was transported to our hospital for further management.

**Figure 1:**
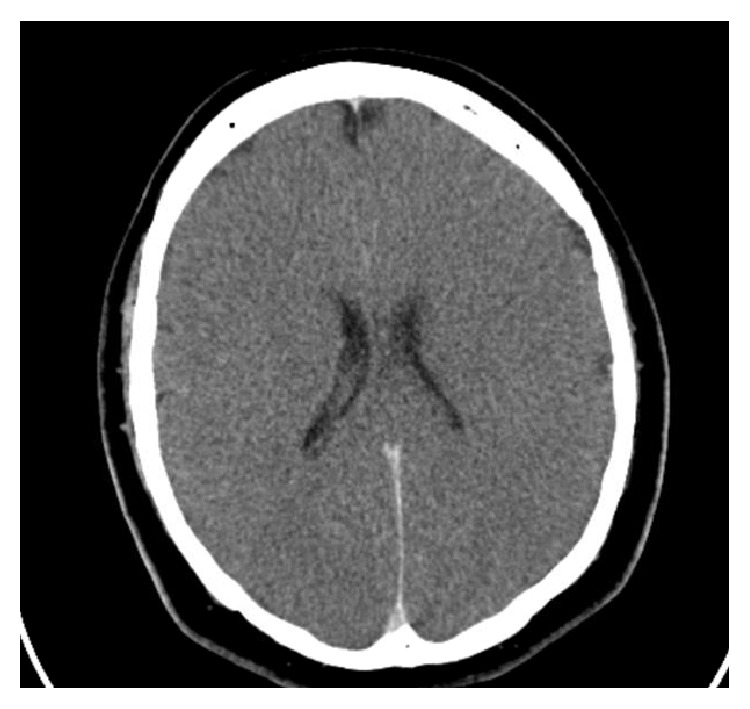
Case #1. Computed tomography of head without contrast with diffuse cerebral edema consistent with brain anoxic injury.

The vital signs were the following: temperature 93.2°F, respiratory rate 16 breaths/minute, pulse 120 beats/minute, blood pressure 115/81 mmHg, and pulse oxygenation 95% on mechanical ventilation. On initial physical exam, pertinent positives include no responsiveness with coarse breaths sounds on ventilator. He had no gag reflex with nonreactive 4 mm pupils. Laboratory tests showed the following: sodium 145 mEq/L, potassium 5.4 mEq/L, chloride 107 mEq/L, bicarbonate 13 mEq/L, blood urea nitrogen 8 mg/dL, creatinine 1.91 mg/dL, and anion gap 32.6. White blood cell count was 12.9 mg/dL, hemoglobin 13.2 mg/dL, and platelet count 106/mm^3^. Arterial blood gas included pH 6.67, carbon dioxide 86 mmHg, oxygen partial pressure 157 mmHg, and bicarbonate 9.7 mEq/L. Hepatic function panel had total bilirubin 0.1 mg/dL, AST 151 u/L, ALT 53 u/L, alkaline phosphatase 74 u/L, creatine kinase 2095 u/L, and ammonia 23 u/L. A lactate level of 16.0 mmol/L was drawn on initial presentation. Urine drug screen was positive for cocaine metabolites, benzodiazepines, and cannabinoids. Chest X-ray showed pulmonary edema. Head computed tomography was performed with diffuse cerebral edema, most consistent with anoxic brain injury (Figure 1).

Brain death protocol was initiated. No neuromuscular or central nervous system medications were administered. The body temperature was found to be within normal limits with maintained systolic blood pressure greater than 100 mmHg. Two neurological examinations showed limited reflexes and failure of the apnea test. The patient was pronounced dead.

## 3. Case Report 2

This patient is a 44-year-old male with a past medical history of spontaneous pneumothorax and urethral stricture who was found unconscious in a metal chrome plating shop for an undetermined duration of time near another unconscious male (Case #1). A bystander discovered the patient and emergency medical services were called. The patient was intubated on the field due to inability to protect airway and decreased mentation. On initial arrival at the emergency department, the patient remained unconscious. Due to the working environment, hydroxocobalamin 5 grams was administered 4 minutes after arrival.

**Figure 2:**
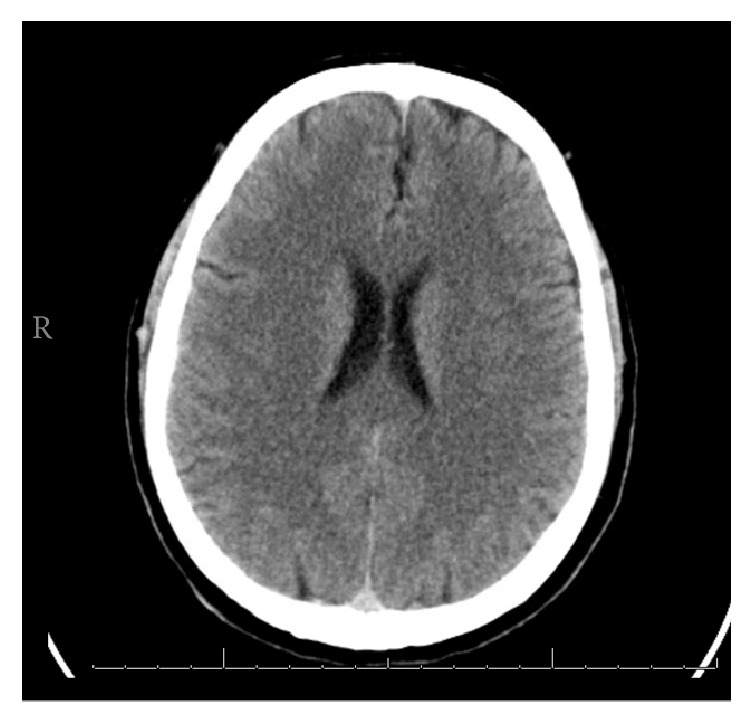
Case #2. Computed tomography of head without contrast with no acute abnormalities.

The vital signs were the following: temperature 97.0°F, respiratory rate 17 breaths/minute, pulse 111 beats/minute, blood pressure 134/80 mmHg, and pulse oxygenation 99% on mechanical ventilation. On initial physical exam in transfer to the intensive care unit (approximately 4 hours after presenting), the patient was awake and responsive to commands. Pupils were equally round and reactive to light and accommodation with extraocular movements intact. Pulmonary exam had coarse breath sounds on ventilator. There was 5/5 strength on upper and lower extremities. Laboratory tests showed the following: sodium 137 mEq/L, potassium 3.5 mEq/L, chloride 99 mEq/L, bicarbonate 9 mEq/L, blood urea nitrogen 12 mg/dL, creatinine 1.85 mg/dL, and anion gap 29. White blood cell count was 16.2 mg/dL, hemoglobin 17.7 mg/dL, and platelet count 283/mm^3^. Arterial blood gas included pH 7.09, carbon dioxide 32 mmHg, oxygen partial pressure 342 mmHg, and bicarbonate 9.0 mEq/L. Hepatic function panel had total bilirubin 0.9 mg/dL, AST 41 u/L, ALT 19 u/L, alkaline phosphatase 91 u/L, creatine kinase 94 u/L, and ammonia 89 u/L. A lactate level of 16.3 mmol/L was drawn on initial presentation. Urine drug screen was positive for cocaine metabolites. Chest X-ray had mild vascular fullness. Head computed tomography was performed with no acute intracranial abnormality and abnormality of the cervical spine (Figure 2).

The patient was given aggressive intravenous fluids. Within 24 hours, the patient was weaned off ventilator settings and extubated. The severe lactic acidosis and metabolic derangements rapidly improved. On the third day, the patient on exam was completely asymptomatic with no residual neurological findings. The laboratory electrolytes were within normal limits. The patient was discharged home.

## 4. Discussion

Cyanide has been observed in toxic exposure for centuries prior to the isolation of the compound [6]. This substance has many potential sources, most commonly from combustion of silk, wool, and synthetic polymers from residential or industrial fires [5, 8]. Cyanide consists of a carbon molecule triple bonded to nitrogen. This compound is highly reactive to metals such as ferric ions [2]. Upon absorption, the compound enters the cellular mitochondria and disrupts cytochrome oxidase a_3_ by binding to the ferric ion [5]. By halting the electron transport chain, adenosine triphosphate production is inhibited leading to anaerobic glycolysis [5].

The absorbed cyanide is primarily metabolized through the liver with an enzyme called rhodanese that catalyzes the conversion of cyanide to thiocyanate. This molecule can be excreted via the kidneys. With large doses of cyanide, this mechanism is overwhelmed largely due to insufficient sulfur donors [2, 5]. A high concentration of cyanide exposure causes sudden inhibition of cellular respiration. Effects can be rapid with possible death resulting within seconds to minutes [5]. With this inhibition, many diffuse clinical symptoms have been observed including dizziness, headache, weakness, tachypnea, diaphoresis, decreased consciousness, and seizures [2, 5]. With no pathognomonic clinical symptom or current rapid cyanide blood test, high clinical suspicion is crucial in the initiation of the cyanide antidote treatments.

Antidote therapy remains integral in the rapid therapeutic intervention that has been previously shown to be effective. Currently, there have been multiple treatments that have been largely evaluated by clinical observation and animal studies [9]. The cyanide antidote kit includes amyl nitrite, sodium nitrite, and sodium thiosulfate [5]. This combination has been used for decades and was demonstrated in our first case. Amyl nitrite has a rapid onset of action and short half-life. Amyl nitrate primarily works by converting hemoglobin to methemoglobin, which binds to cyanide and allows cytochrome oxidase a_3_ to reactivate the electron transport chain [5, 9]. Additionally, vasodilation occurs through nitrites which may also provide a protective benefit from cyanide toxicity [5].

Sodium thiosulfate is given in combination with nitrites and has shown improvement in survival in animal studies [9]. Additionally, with no clinical trials, the effectiveness has been extrapolated from case reports [10]. Sodium thiosulfate acts as a sulfur donor in the conversion of cyanide to thiocyanate through rhodanese [11]. It has poor penetration into the mitochondria, which is the site of action, causing slow onset [9]. With a short half-life and delay onset, sodium thiosulfate must be given in combination with other therapies [5]. The primary concern in this administration of the cyanide antidote kit is the side effects, including severe hypotension, methemoglobinemia, and hypersensitivity reactions [2]. Additionally, although methemoglobin will alleviate the cyanide burden on aerobic metabolism, the resulting methemoglobinemia will decrease the ability of the red blood cells to release oxygen to tissues [2].

Hydroxocobalamin has been used in treatment of cyanide poisoning for over 30 years, with gradually expanding acceptance of its safety and effectiveness [2]. Cyanide has a greater affinity to bind with hydroxocobalamin rather than cytochrome oxidase a_3_ forming cyanocobalamin [2, 3]. This newly formed compound is renally excreted [12]. The effectiveness has been shown in retrospective and prospective studies. Additionally, animal studies have displayed improved mortality with treatment [6]. Side effects have been considered more minimal with findings of headaches, hypersensitivity reactions, hypertension, reflex bradycardia, reddening of the skin, and urine discoloration [3, 11]. With a history of less severe adverse reactions and acute onset, this cyanide antidote treatment has been gaining acceptance [12].

The effects of cyanide poisoning are rapid with recovery determined by the timing of exposure and administration of treatment. Presently, both cases treatments were administered in the emergency department upon arrival. These antidotes do not appear on the formulary for the emergency medical services that arrive at the scene of exposure. This could be related to the prior cyanide antidote kit containing sodium nitrate and amyl nitrite which both can cause methemoglobinemia. In the most common setting of cyanide inhalation with residential fires and no rapid cyanide diagnostic test, administration without true cyanide exposure would inadvertently harm the victim. With the limited side effects from hydroxocobalamin, this could at the present time be reconsidered.

In this report, we have two cases of cyanide poisoning from the same source that were treated with the two most available and accepted antidotes. There were no available studies to confirm the extent of the exposure. Based on clinical findings, Case #1 likely had more severe exposure with sudden pulseless electrical activity and subsequent requirement of vasopressors. There have been swine animal studies comparing the effectiveness of both antidotes on acute cyanide toxicity. In animal studies and case reports, combination therapies showed potential synergist effect when combining sodium sulfate and hydroxocobalamin [13, 14]. A 2012 study by Bebarta et al. performed on swine found sodium thiosulfate failed to reverse cyanide-induced shock and hydroxocobalamin was found to be effective. A combination of therapies did not further improve the outcome [6]. In 2015, a case report combined both treatments following potassium cyanide ingestion which resulted in favorable results without long-term neurological sequelae [15]. Presently, further studies are required to compare the effectiveness of the two present cyanide treatments. In the current cases, both treatments were separately used with favorable results following hydroxocobalamin. The more clinically severe case was treated with the combination therapy of sodium thiosulfate and nitrates. This remains a difficult assessment with the patient previously having pulseless electrical activity prior to cyanide treatment. Even with hydroxocobalamin treatment, it could have possibly had the same end result with the rapid nature of cyanide toxicity. More studies are required to determine the overall effectiveness of treatment when comparing the two available cyanide antidotes.

## 5. Conclusion

Cyanide poisoning has two present treatments that have been proven effective. Hydroxocobalamin has been shown to decrease mortality with less severe associated side effects in comparison to sodium thiosulfate. The present cases illustrated the effectiveness of hydroxocobalamin resulting in no neurological sequelae and another case resulting in death following sodium thiosulfate treatment. With decreased side effects, hydroxocobalamin availability for emergency medical services for rapid administration should be reconsidered for increased effectiveness for reversible therapy.
